# How biological sex shapes differences in immune responses to infection

**DOI:** 10.1186/s13293-026-00881-1

**Published:** 2026-05-12

**Authors:** Megan J. Wilson, Amedeo Amedei, Pora Kim, DeLisa Fairweather

**Affiliations:** 1 Department of Anatomy, Faculty of Biomedical Sciences, University of Otago, Dunedin, 9054 New Zealand; 2 Department of Experimental and Clinical Medicine, University of Florence, Florence, 50134 Italy; 3 Center for Computational Systems Medicine, McWilliams School of Biomedical Informatics, The University of Texas Health Science Center at Houston, Houston, TX 77030 USA; 4 Department of Cardiovascular Medicine, Mayo Clinic, Jacksonville, FL 32224 USA

## Introduction

Biological sex influences virtually every aspect of immune function, from infection susceptibility and vaccine responses to autoimmunity and cancer immunotherapy. Yet, despite decades of observation that males and females differ in disease outcomes, our mechanistic understanding of these differences remains incomplete. Emerging research reveals that sex differences in immunity arise from layered interactions between sex chromosome dosage, hormone signalling across the lifespan, epigenetic regulation, and tissue-specific immune cell states. At the same time, social and environmental factors intersect with biology, complicating interpretation and translation to treatment.

### Beyond hormones - sex chromosome regulation of immune function

Growing evidence indicates a role for the X chromosome in promoting inflammation in diseases that occur more often in females, such as autoimmune diseases. Because females have two X chromosomes (46XX), one X chromosome is randomly inactivated in each cell by the long non-coding RNA (lncRNA) X-inactive specific transcript (*XIST*). This process helps to equalize X-linked gene expression between males and females. However, around 15%-23% of X-linked genes escape X chromosome inactivation, resulting in higher expression of proteins encoded by those genes in females. This difference in gene dosage can influence immune responses and may contribute to sex-specific differences in responses to infection (as reviewed in) [1–3]. Several genes that are relevant in mediating immune responses to infection that are found on the X chromosome include forkhead box P3 (*FOXP3*), which inhibits inflammation, interleukin (IL)-2 receptor gamma chain (*IL2RG*), important for T, B and natural killer cell function, Toll-like receptor (*TLR*)*7* and *TLR8*, relevant in innate immune function, cluster designation ligand (*CD40L*), crucial in T cell activation, Bruton’s tyrosine kinase (*BTK*), critical in B cell activation, and C-X-C motif chemokine receptor 3 (*CXCR3*) which is involved in immune cell migration during inflammatory responses [4, 5]. In addition, lncRNA *XIST* has been shown to enhance TLR7-mediated immune activation, which correlates with higher interferon (IFN) signatures and disease activity in females with systemic lupus erythematosus (SLE) [6]. Sex hormones and X chromosome-linked genes are not the only contributors to sex differences in the immune response following infection; evidence indicates that epigenetic programming strongly influences immune responses.

### Epigenetic programming, non-coding RNAs and long-lived immune memory

Epigenetic programming, including DNA methylation, histone modifications, and microRNA (miRNA)-mediated regulation, may contribute to sex differences in immune function. MicroRNAs are short, single-stranded, noncoding RNAs that form complementary base-pairs with the 3′ untranslated region of target mRNAs within the RNA-induced silencing complex (RISC). They trigger mRNA degradation and translational inhibition, ultimately reducing both mRNA expression levels and protein production [7]. MicroRNAs can be free-floating in the cell or released into the circulation in extracellular vesicles (EVs), allowing them to influence gene regulation in other cells.

The human genome contains approximately 2,500 mature miRNAs, predicted to regulate an estimated 60% of protein-coding mRNAs [8]. The X chromosome is enriched for miRNAs, encoding approximately 10% of all known miRNAs [9]. As miRNAs regulate many immune-related genes, this enrichment is one potential mechanism underlying sex differences in immune responses and the higher incidence of autoimmune diseases in females.

Sex-specific epigenetic differences in immune regulation can emerge early in life. A recent study deciphering immune-related DNA methylation patterns in whole blood from the Canadian Healthy Infant Longitudinal Development (CHILD) cohort (*n* = 760) revealed widespread sex-specific differences across multiple immune cell types at ages one and five [10]. These findings suggest that sex-biased epigenetic regulation of immune function is established well before puberty.

### Hormonal driven sex differences in the immune system

Sex differences in immune responses are partly driven by the effects of sex hormones on many immune cells. Estrogen receptors are expressed on many immune cell types, including T cells, B cells, monocytes, macrophages, and dendritic cells, allowing estrogen to modulate immune signalling and antibody production [1, 11]. In general, estrogen enhances immune activiation and antibody (and autoantibody) levels in females by inhibiting apoptosis, and enhancing cell differentiation and antigen presentation (as reviewed in)[1, 12, 13]. Whereas, the imunological effects of testosterone are less well understood. Thus, together with genetic differences arising from the X chromosome, hormonal effects contribute to sex differences in immune cell function and sex-biased responses to infection.

### Sex differences following infection or vaccination

Sex differences in immune responses are also evident following vaccination and infection. Females typically develop stronger antibody responses after vaccination, which may enhance protection from infection, but they often report more side effects than males [1, 14]. In contrast, men frequently experience more severe symptoms and higher mortality rates from infections, such as SARS-COV-2 infection. However, females are more susceptible to prolonged post-viral conditions such as Long-COVID) [1, 14].

Several genes involved in antiviral immunity, including *IFNG* and interferon regulatory factor 5 (*IRF5*), contain estrogen response elements, suggesting that estrogen signalling may enhance antiviral immune response in females and contribute to better control of infections such as influenza and SARS-COV-2 [1, 12, 15]. Consistent with this, males are more likely to develop viral myocarditis and COVID-19 mRNA vaccine-induced myocarditis [13]. Experimental models further illustrate these sex differences. Following coxsackievirus B3 infection of mice, both sexes clear the virus but females are protected from severe cardiac outcomes through immune regulatory mechanisms that limit inflammation [13]. These are just a few examples of the numerous sex differences in the immune response following infection or vaccination.

### Microbiome sex differences and infection

Another relevant source of immune regulation to infection occurs in the gut microbiome. In detail, it has been proposed that the microgenderome contributes to sex-based differences observed in chronic inflammatory conditions such as autoimmune diseases. The microgenderome is defined as the gender- or sex-specific composition of the human microbiome shaped by sex hormones, which, in turn, regulates the immune response [16].

The microgenderome is a critical factor in determining how host immunity recognizes and distinguishes pathogens. Men and women display distinct microbial profiles in the gut, skin, and urogenital tracts, largely driven by the bidirectional relationship between sex hormones and bacterial diversity [17] For example, estrogen can promote the growth of specific beneficial bacteria while modulating the gut barrier’s integrity, often resulting in females mounting a stronger immune response to viral and bacterial invaders than males. In addition, high estrogen levels promote glycogen accumulation in the vaginal lining. Lactobacillus species ferment this glycogen into lactic acid, maintaining a low pH (around 3.5–4.5). This acidic environment is a formidable barrier against pathogens that cause urinary tract infections (UTIs) and sexually transmitted infections (STIs). On the contrary, males lack this *Lactobacillus*-dominant defence, resulting in a different and less acidic urogenital microbiome. While men get fewer UTIs due to anatomy, their microbiome composition can influence the persistence of certain viral infections [18].

Remarkably, the microgenderome is not limited to reproductive organs but extends to other host niches, including the lungs, via the gut-lung axis, influencing the local susceptibility to viral and bacterial infections. Studies focusing on influenza A and SARS-CoV-2 have shown that gut microbial diversity, which is higher in females due to estrogen, can prime the systemic immune response more effectively [19, 20]. Finally, other reports suggest that some gut bacteria, influenced by sex hormones, produce metabolites (such as short-chain fatty acids) that circulate through the bloodstream to the lungs. These metabolites may enhance alveolar macrophages’ ability to clear bacterial pathogens [21, 22]. Understanding how the microgenderome influences sex differences in the immune response to infection moves researchers and clinicians away from “one-size-fits-all” medicine toward more personalized, sex-aware treatments for infectious diseases.

### Emerging technologies, research and policy gaps

Despite strong evidence that biological sex fundamentally shapes immune function, key research and policy gaps remain in understanding mechanisms and translating insights into clinical practice. First, sex has specific effects across the lifespan [23, 24], indicating that reporting and analyzing data by sex alone is insufficient. Data needs to be thoughtfully reported and analyzed by sex and age, which should be incorporated into future policy changes. Secondly, advances in single-cell and spatial multi-omics technologies offer unprecedented resolution for dissecting sex-specific immune cell states and their interactions with tissue. Single-cell multi-omics platforms integrating transcriptomic, epigenomic, and proteomic data can capture cellular heterogeneity and regulatory networks underlying divergent immune responses in males and females across health and disease contexts. For example, a recent review highlights how these methods uncover cellular and molecular complexity that bulk assays miss, enabling precision interrogation of immune dysregulation in sex-biased diseases such as autoimmunity and cancer [25]. Thus, preclinical and clinical data must be analyzed by sex and age, rather than by combining males and females and age groups, for insights to be gained from these techniques.

The need for analysis by *sex and age* is even more relevant with the advent of capabilities to analyze bulk data from data repositories. If the data is not deposited disaggregated by sex and age, deep learning models and artificial intelligence tools will not have the data provided in the manner that is needed to perform analysis according to sex and age- leading to many erroneous conclusions [26]. There is an urgent need to start policy changes to verify that data has been deposited to sites disaggregated by sex and age. Another key research and policy gap is that clinical trials rarely incorporate hormonal status, contraceptive use, pregnancy, or menopausal status into analyses, obscuring possible mechanistic drivers of disease in women. Mandating sex- and age-disaggregated data reporting and inclusion of endocrine variables in study protocols would improve reproducibility and therapeutic relevance and should be included in future policy initiatives.

And finally, before knowledge of sex-based immunological effects on infections can be translated into targeted and effective therapies, collaborative networks that bridge basic and translational science, clinical trials, and regulatory policy are needed to ensure mechanism-informed interventions and therapies tailored to sex and age. To bridge the gap between these disparate biological layers, we may need to develop a comprehensive foundation model for sex and age-biased immunity. By integrating large-scale multi-omics with longitudinal clinical data, such a model can capture the non-linear interactions between X-chromosome dosage, hormonal fluctuations, and microbial diversity. This computational framework will move beyond traditional disaggregated analysis, providing a predictive engine to realize truly personalized, sex-age-aware precision interventions.
